# Microencapsulation of Deer Oil in Soy Protein Isolate–Chitosan Complex Coacervate—Preparation, Characterization, and Simulated Digestion

**DOI:** 10.3390/foods14020181

**Published:** 2025-01-09

**Authors:** Hongyan Li, Ying Zong, Weijia Chen, Yan Zhao, Jianan Geng, Zhongmei He, Rui Du

**Affiliations:** 1 College of Chinese Medicinal Materials, Jilin Agricultural University, Changchun 130118, China; lhyok0726@163.com (H.L.); zongying7699@126.com (Y.Z.); chenweijia_jlau@163.com (W.C.); zhaoyan@jlau.edu.cn (Y.Z.); gengjianan@jlau.edu.cn (J.G.); 2 Jilin Provincial Engineering Research Center for Efficient Breeding and Product Development of Sika Deer, Jilin Agricultural University, Changchun 130118, China; 3 Key Laboratory of Animal Production and Product Quality and Safety, Ministry of Education, Jilin Agricultural University, Changchun 130118, China

**Keywords:** soya protein isolate, chitosan, complex coacervate, microencapsulation, deer oil

## Abstract

Deer oil (DO) is a potentially beneficial functional oil; however, its sensitivity to environmental factors (e.g., oxygen and heat), difficulty in transport, and unfavorable taste hinder practical use. In this study, DO was encapsulated through the cohesive action of soy protein isolate (SPI) and chitosan (CS). The optimal preparation conditions yielded microcapsules with DO’s highest encapsulation efficiency (EE) (85.28 ± 1.308%) at an SPI/CS mixing ratio of 6:1 and a core-to-wall ratio of 1:2 at pH 6. Fluorescence and scanning electron microscopy were utilized to examine the microcapsules’ structure, showing intact surfaces and effective encapsulation of oil droplets through SPI/CS composite coalescence. Through Fourier transform infrared spectroscopy (FTIR), the electrostatic interplay between SPI and CS was verified during the merging process. At room temperature, the microcapsules resisted core oxidation by reducing gas permeation. In vitro simulated digestion results indicated the microcapsules achieved a slow and sustained release of DO in the intestinal tract. This study further expands the application scope of deer oil and promotes the development of deer oil preparations and functional foods.

## 1. Introduction

Deer oil (DO) is the fatty oil of the sika deer (*Cervus nippon*) or horse deer (*Cervus elaphus*), also known as deer fat. It is a natural biological resource from a wide range of sources, easy to obtain, has many health functions for human beings, and is a standard functional food oil. DO is recorded in the ancient Chinese book *Tang Materia Medica*: “Lord of carbuncle, swelling, and dead muscle, warming the middle, limbs do not follow the wind, head, and pass the coupling.” It has the effect of detoxification and swelling reduction. Some scholars have shown that DO has specific antioxidant and anti-inflammatory activities and can produce significant protective benefits against ethanol-induced acute gastric mucosal damage [1]. Deer oil also demonstrated significant pre-protective effects against ulcerative colitis in mice induced by dextran sulfate sodium [2]. Thus, deer oil is a potentially beneficial functional oil that is socially important in meeting the needs of different consumer groups. However, fatty acids may degrade during food processing and storage, causing a loss in flavor and nutritional quality due to their high degree of unsaturation. Additionally, deer oil’s sensitivity to environmental factors (e.g., oxygen, light, and heat), the difficulty of weighing for transport, and the unfavorable flavor are reasons why it cannot be used effectively. Microencapsulation of fatty oils has received significant attention due to its ability to optimize product performance, facilitate mass production, reduce transport costs, and protect nutrients [3]. Concealing the natural taste and smell characteristics in food formulations is sometimes as crucial as their chemical stability. Microencapsulation can hide the intense flavors and tastes of deer oils, while also preventing oxidation and promoting their controlled release.

A popular strategy for improving the storage stability, functional qualities (poor bioavailability), and physical characteristics (unpleasant taste and flavor) of active chemicals is microencapsulation, which creates a physical barrier between the encapsulated element and its environment [4]. Furthermore, given the right circumstances, the encapsulating substance can be freed [5,6]. The technique of complex coacervation stands out as an efficient way to create microcapsules that enclose essential oils, functional oils, vitamins, carotenoids, and anthocyanins. Its benefits include gentle processing environments, substantial payload capacity, and superior encapsulation efficiency [7]. The use of complex coacervate is extensive in encapsulating oleaginous elements like hemp seed oil [8], chia seed oil [9], and algal oil [10]. Complex coacervation primarily arises from the electrostatic pull between two biopolymers with opposite charges [11]. The complex coacervate frequently employs proteins and polysaccharides as wall substances to create microcapsules [12,13]. The complex coalescence of polysaccharides and proteins primarily occurs through electrostatic interactions. When oppositely charged polysaccharides and proteins are combined in solution, they attract one another, resulting in the formation of complexes. These complexes can subsequently coalesce to yield larger aggregates. The specific stages involved in this process include initial mixing, electrostatic interactions, complex formation, and coalescence. Ultimately, under suitable conditions, these complexes amalgamate into larger aggregates, which have diverse applications in food science and biomedical fields.

Proteins interact well with solvents and encapsulated substances because they are amphiphilic biomolecules [14]. Soya protein isolate (SPI) is a major plant-based protein, primarily consisting of 11S (glycine) and 7S (β-co-glycine) globulins. These proteins are highly valued due to their natural availability, excellent emulsifying abilities, good solubility in water, and well-rounded amino acid composition, which supports beneficial interactions with other nutrients [15]. Therefore, SPI is an ideal wall material for DO microencapsulation. A common linear cationic polysaccharide for the administration and regulated release of functional chemicals is chitosan (CS) [(1-4)-2-amino-2-deoxy-β-d-glucan]. Chitosan has the advantages of low toxicity, biocompatibility, biodegradability, and antioxidant properties, and it has been shown that microcapsules can be formed by dropping a chitosan solution into an anionic solution through complex coacervation [16,17,18] and protects venison oil from oxidation. The delivery systems created from various proteins and polysaccharides differ significantly in structure and properties. Factors such as temperature, pH, and the polysaccharide/protein ratio during construction greatly influence the performance of these systems [19]. There have been numerous studies on SPI and CS complexes and their application in delivery systems. These complexes strongly stabilize emulsions [20,21] and improve the preservation of bioactive molecules through microencapsulation [22]. However, the microencapsulation of deer oil using the cohesive forces of SPI/CS complexes has not been documented; this research intends to explore the interaction between SPI and chitosan, focusing on the microencapsulation of deer oil with SPI/CS complexes as the encapsulant. The interaction between SPI and CS coagulant was assessed, with a focus on the formation of liquid microcapsules, their physical characteristics, and oxidative stability. This research could offer insights into the possible application of SPI and CS for microencapsulating deer oil.

In this study, we investigated the optimal ratio of SPI to CS complex coacervation and optimal pH. Techniques such as Fourier transform infrared (FTIR) analysis and scanning electron microscopy were used to examine the chemical and physical characteristics of the SPI/CS complexes, as well as to observe the release behavior of deer oil-loaded microcapsules in a simulated gastrointestinal environment. This research sought to explore how various factors influence the creation of SPI/CS coacervates and to clarify the interaction mechanism between SPI and CS, potentially offering useful insights for the development of new functional food ingredients utilizing DO encapsulated in SPI/CS coacervates.

## 2. Materials and Methods

### 2.1. Materials

Soya protein isolate, product number S30914, was obtained from Shanghai Yuan Ye Company. Chitosan (viscosity average molecular weight of 150 kDal and ≥75% deacetylation, molecular formula (C_6_H_11_NO_4_)_n_), product number C299272, was from Shanghai Aladdin Reagent Co., Ltd. (Shanghai, China). The deer oil used was unprocessed fresh Meihua deer plate oil from Changchun Deer Town Huatai Biotechnology Co., Ltd. (Changchun, Jilin Province, China). The transglutaminase (EC 2.3.1.13) was supplied by Taixing Yiming Biological Co., Ltd. (Taixing, Jiangsu, China). Pepsin (>3000 U/mg, product number P6322) was obtained from Shanghai Macklin Biochemical Co., Ltd. (Shanghai, China). Trypsin (>250 U/mg, product number BS130) was purchased from Labgic Technology Co., Ltd. (Hefei, China).

To produce simulated gastric fluid (SGF) [23], the procedure entailed dissolving 1.0 g of pepsin in 80 mL of ultra-pure water, followed by adjusting the pH to 1.2 using a 10% hydrochloric acid solution. Subsequently, the mixture was thinned down to 100 mL and passed through a sterile 0.22 μm filter membrane for application.

To prepare the simulated intestinal fluid (SIF) [24], 0.1 g of trypsin and 0.3 g of bile salts were dissolved in 100 mL of 0.2 mol/L phosphate buffer at pH 6.8, and the solution was then filtered through a 0.22 μm sterile membrane.

### 2.2. Extraction of Deer Oil

The extraction of deer oil was performed using the digestive boiling technique: take fresh deer plate oil; remove attached hair, skin, and other impurities and debris; rinse, drain, and cut into small pieces; place the pieces into a small amount of water, then heat to 120 °C until the water evaporates; then boil out the grease after filtering off the dregs of vacuum drying, thus yielding deer oil.

### 2.3. Gas Chromatography–Mass Spectrometry (GC–MS) Analysis of Fatty Acids in DO

The DO was subjected to methyl esterification of fatty acids. For sample preparation, 0.2000 g of extracted DO was precisely measured and dissolved in n-hexane, then brought to volume in a 10 mL volumetric flask. The solution was shaken well, and 50 μL was pipetted into a 10 mL test tube. Subsequently, 2 mL of a 0.4 mol/L hydroxide–methanol solution was carefully introduced, and the resulting mixture was subjected to vortexing for 10 min to ensure thorough mixing. Following a 10 min incubation, 1.95 mL of n-hexane was introduced, and the sample underwent vortexing for an additional 2 min. The resulting mixture was then diluted to a final volume of 10 mL using an 8% (*w*/*v*) NaCl solution, followed by centrifugation at 800× *g* for 10 min. The supernatant was carefully collected into a microtube, and 1 μL of this solution was then analyzed using GC-MS [25].

The chromatographic conditions employed a DB-5MS capillary column (30 m × 0.25 mm × 0.25 μm), with the inlet temperature set at 250 °C. The mass spectrometry conditions comprised an EI+ ion source operating at 70 eV, with a filament current of 0.2 mA, an ion source temperature of 230 °C, and an interface temperature of 250 °C, covering a mass scanning range from 10 amu to 550 amu. Spectral analysis was carried out through qualitative identification based on retention time and library matching, followed by quantitative comparison with appropriate standards to determine the primary chemical constituents of DO.

### 2.4. Plotting of Deer Oil Standard Curves

**Figure 1:**
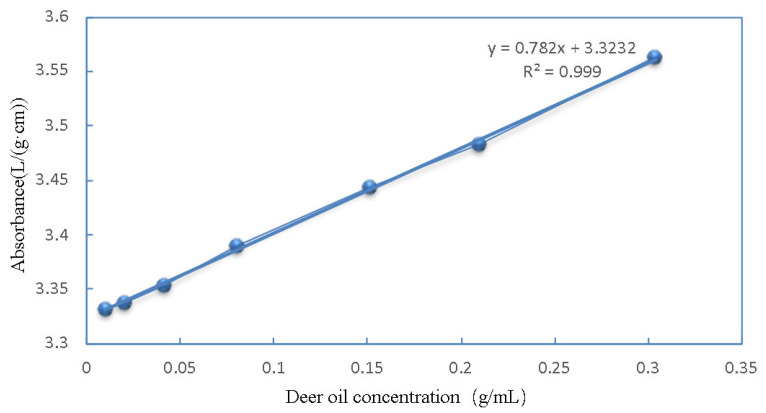
Standard UV curve of deer oil.

With n-alkane as the blank reference, ultraviolet (UV) scanning was carried out at 200~400 nm, and it was determined that the n-alkane solution of deer oil had the maximum absorption at 219 nm, so 219 nm was chosen as the UV detection wavelength of deer oil. Deer oil solution was diluted to the standard series of mass concentration (10, 20, 40, 80, 150, 210, 300 mg/mL), with n-hexane as a blank, and the maximum absorption wavelength for deer oil was determined to be 219 nm, based on the absorbance (A) value of the standard solution. The A value for the vertical coordinates and the deer oil mass concentration as the horizontal coordinates (C) were used to draw the deer oil standard curve (Figure 1), and the regression equation for A = 0.782C + 3.3232, with R^2^ = 0.999. The findings indicate that deer oil exhibited good linearity within the concentration range of 10–300 mg/mL [26].

### 2.5. Preparation of SPI, CS Complexes, and Microcapsules

#### 2.5.1. Preparation of SPI and CS Complexes

A 1% (*w*/*v*) chitosan solution was prepared by dissolving chitosan powder in a 1% (*v*/*v*) acetic acid solution, followed by magnetic stirring for 3 h at room temperature. A 1% (*w*/*v*) soya protein isolate stock solution was prepared by dissolving the protein in ultrapure water heated to 40 °C, then stored at 4 °C until needed.

The 1% (*w*/*v*) soy isolate protein solution and 1% (*w*/*v*) chitosan were mixed in different ratios (1:1, 2:1, 3:1, 4:1, 5:1, 6:1, 7:1, 8:1, and 9:1). The composite solution was heated to 45 °C for 30 min, and its pH was subsequently adjusted with 5 M HCl and NaOH to the following values: 2.0, 2.5, 3.0, 3.5, 4.0, 4.5, 5.0, and 5.5, leading to the formation of the complex condensate. The optimization of pH and ratios was achieved by measuring zeta potential, electro-static interaction strength, turbidity, and yield.

#### 2.5.2. Preparation of Deer Oil Microcapsules

**Figure 2:**
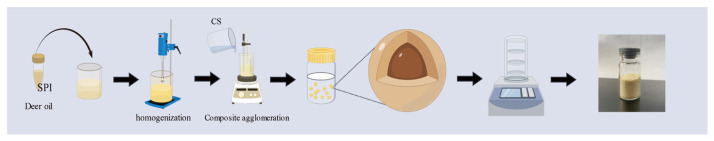
Diagram illustrating the preparation process of deer oil microcapsules.

Microcapsules were synthesized via emulsification based on the procedure described by Hu et al. [27], followed by freeze-drying. Deer oil was emulsified by adding it to the soybean isolate protein stock solution. The mixture was subjected to homogenization at 10,000 rpm for 2 min with a high-speed homogenizer (IKA; Guangzhou, China), and this process was repeated twice. The prepared emulsion was heated to 45 °C, followed by the dropwise addition of the chitosan stock solution. The pH was modified using a 10% NaOH solution to facilitate the coagulation of the soy protein isolate–deer oil–chitosan complex. The stirring rate was 300 rpm, and the pH was adjusted to 6 to promote the coalescence of the complex, which was kept for 30 min. Glutamine transaminase (TGase) was added at 30 U/g soybean isolate protein for curing deer oil microcapsules. The microcapsules were kept at 25 °C for 6 h, with continuous stirring at 300 rpm to allow for complete cross-linking. Afterward, the samples were incubated overnight at 4 °C, followed by centrifugation at 800× *g* for 5 min. The resulting precipitate was gathered and subjected to freeze-drying to yield the final SPI-DO-CS microcapsules (Figure 2).

### 2.6. Detection of SPI, CS Complexes, and Microcapsules

#### 2.6.1. Zeta Potential, Static Electric Interaction (SEI), Turbidity, Yield Detection

The 2% (*w*/*v*) stock solution was diluted to 0.1% (*w*/*v*), and the pH of the resulting composite solution was adjusted with 5 M HCl and NaOH to values of 2.0, 2.5, 3.0, 3.5, 4.0, 4.5, 5.0, and 5.5. The ζ potential was determined with a Malvern ZEN 3600 laser particle size analyzer (Malvern; Malvern City, UK). The electrostatic interaction strength (SEI) was determined using Equation (1) as shown below.

SEI (mV^2^) = ζ of soybean isolate protein × ζ of chitosan (1)

Turbidity measurements of the complexes were performed at 600 nm with a UV–vis spectrophotometer (UV-1780, Shimadzu; Kyoto, Japan).

After the SPI/CS mixture was left undisturbed for 10 min, it was carefully transferred into a 1 cm cuvette without stirring. Turbidity was immediately assessed at 600 nm with a spectrophotometer (Unico, UV 2000; Shanghai, China).

The amount of complex coacervate was quantified by centrifuging the coagulum solution at 800× *g* for 20 min. The precipitate was then collected and lyophilized. The yield was calculated from Equation (2) as follows.

(2) $$\mathrm{Yield}\;(\%)=\frac{m_{1}}{m}\times 100\%$$

Here, $m_{1}$ represents the weight of the condensed complex after freeze-drying, and *m* denotes the mass of total solids before complex coacervation.

#### 2.6.2. FTIR

The chemical structure of complex coacervates and microcapsules was determined using the Vertex70 FT-IR Spectrometer (Bruker; Saarbrücken, Germany) with an attenuated total reflectance (ATR) accessory. The spectrum was recorded within the wavenumber range of 400–4000 cm^−1^ at a resolution of 4 cm^−1^.

#### 2.6.3. Measurement of Embedding Rate

The Soxhlet extraction method was used to determine the total oil content [28]. First, 1 g of microcapsules was weighed into a filter paper tube and placed in the extraction tube. The lower part of the extraction tube was connected to the receiving bottle and the upper part was connected to the condenser tube. Petroleum ether was added to the receiving bottle. The receiving bottle was put in a water bath to heat up, so that the petroleum ether kept refluxing and extracting, and the receiving bottle was removed after 8 h. The solvent was then removed by rotary evaporation. Then, the sample was subjected to drying at 105 °C until a stable weight was reached, enabling the determination of the total oil content in the microcapsules.

The process was as follows. Weigh 1 g of microcapsules in a conical flask, add 20 mL of petroleum ether, and shake for 30 s; then use filter paper to filter and wash the filtrate with 10 mL of petroleum ether several times, combine the filtrate, recover the solvent by rotary evaporator, and put it into an oven to evaporate the solvent at 105 °C. Transfer the weighing bottle to a dryer and let it cool down. The sample was then weighed to determine the surface oil content of the microcapsules. The weighing bottle was placed in a desiccator and weighed once cooled to determine the surface oil content of the microcapsules [28].

The microcapsule embedding rate is calculated as shown in Equation (3).

(3) $$\mathrm{EE}\%=\frac{T_{0}-S_{0}}{T_{0}}\times 100\%$$

where EE, embedding rate; T_0_, total oil content; S_0_, surface oil content.

#### 2.6.4. Microstructural Observations

Fluorescence staining was employed to ascertain the inner composition of the microcapsules. Newly made microcapsule suspensions underwent staining using a combined fluorescent solution containing 1.0 mg/mL Nile Red and 1.0 mg/mL fluorescein isothiocyanate (FITC). Following this, 3.0 μL of the dyed microcapsule mixture was moved onto a solitary concave slide, and the structure of a small sample of the prepared microcapsules was observed with a Nikon 80I fluorescence microscope (Nikon; Tokyo, Japan).

#### 2.6.5. Scanning Electron Microscopy (SEM)

A scanning electron microscope (JEOL JSM 6390 LV, Singapore) was employed to scrutinize the microstructure of freeze-dried microencapsulated powders. After drying, the microencapsulated powder underwent sputtering and platinum coating, followed by capturing images at a 5 kV accelerating voltage and a magnification of 2000×. Scanning electron microscopy (SEM) visuals were captured from the surface layer of the microencapsulated powder [29].

#### 2.6.6. Differential Scanning Calorimetry (DSC)

The Differential Scanning Calorimeter (DSC822E, Perkin–Elmer; Waltham, MA, USA) was used to measure DSC. A precise measurement of 5 mg from the samples was taken before their placement in a crucible. A hollow crucible served as the benchmark. The temperature ranged from 30 to 300 °C, with a heating rate of 10 °C per minute.

#### 2.6.7. Physical Property Testing

The particle size distribution and polydispersity index (PDI) of particles in a solution were quantified using the dynamic light scattering mode of the Malvern laser particle size analyzer (ZEN 3600, Malvern; Malvern, UK). In a water-filled measuring chamber, microcapsule powder was mixed to achieve a 20% level of concealment. The integrated software documented the dimensions and spread of the particles.

Around 1 g of each microcapsule sample was measured and heated in an oven at 105 °C until the weight stabilized. The moisture content was determined by dividing the loss in weight during drying by the sample’s initial weight.

The technique outlined by de Barros Fernandes et al. [30] was used to ascertain hygroscopicity. In summary, the microencapsulated specimens (around 1 g) underwent drying at 105 °C in an oven until they reached a stable weight. After drying, the microencapsulated samples were measured and placed into a desiccator containing a saturated NaCl solution, ensuring 75% relative humidity at 30 °C. Following a period of 7 days, the samples underwent weighing, and their hygroscopicity was determined by the proportion of the weight variance between the samples pre- and post-hygroscopicity and the weight of the dried microencapsulated specimens.

Microcapsule bulk density was assessed using a revised method adapted from Zhang et al. [31]. A 2 g portion of microencapsulated powder was placed into a 10 mL test tube. The bulk density was determined by calculating the ratio of the powder’s mass to the volume it displaced within the test tube.

Around 2 g of powder was placed into a 10 mL graduated cylinder and lightly compressed. Bulk density ($\rho_{bulk}$) was calculated by dividing the powder’s mass by the volume it occupied in the cylinder. To determine tapped density ($\rho_{tapped}$), about 5 g of powder was placed into a 25 mL volumetric cylinder. The sample was gently compressed by alternating raising and lowering the cylinder until the powder settled to a consistent level. Tapped density was determined by dividing the powder mass by its volume after being compacted in the cylinder.

The flow characteristics and powder sample cohesiveness were assessed using Carr’s index (CI) and the Hausner ratio (HR), in accordance with the formulas given in Equations (4) and (5), respectively.

(4) $$\mathrm{CI}=\frac{\rho_{tapped}-\rho_{bulk}}{\rho_{tapped}}\times 100$$

(5) $$\mathrm{HR}=\frac{\rho_{tapped}}{\rho_{bulk}}$$

The experiment commenced by releasing the funnel outlet, permitting the powder to flow freely and form a cone on the surface. A funnel was positioned 10 cm above a flat plane. The procedure started with the release of the funnel outlet, allowing the powder to flow freely, forming a cone on the surface. The repose angle (θ) was calculated using the height (h) and radius (r) of the powder cone, as outlined in Equation (6) [31].

(6) $$\mathrm{Tan}\theta =\frac{h}{r}$$

### 2.7. Determination of Oxidative Stability of Deer Oil Microcapsules

The freeze-dried microencapsulated powder was kept at 37 °C for a period of 5 weeks. Throughout this time, samples were taken every week for additional examination. Lipid peroxidation was evaluated using the procedure outlined by Chen et al. [32]. The concentration of reactive oxygen species in a 1.0 kg sample was determined as the peroxide value. Each test involved preparing at least three distinct samples, with three measurements taken for each.

### 2.8. In Vitro Simulated Digestion of Microcapsules

The digestive behavior of the microcapsules was assessed using an in vitro model that included both gastric and intestinal fluids [33]. The release kinetics of deer oil in simulated gastrointestinal fluid were assessed by subjecting the microcapsules to simulated gastric fluid for 2 h, then incubating in SIF for 4 h. Specifically, 1 g of deer oil microcapsule powder was added to 20 mL of gastric fluid substitute. The pH was adjusted to 2.0, and the mixture was placed on a shaker for agitation and incubated at 37 °C with a shaking speed of 100 rpm to mimic gastric digestion for 0 to 2 h. Throughout this duration, 2 mL of digestive fluid was withdrawn every 30 min, with an equivalent volume of simulated gastric fluid added to maintain the fluid volume. After 2 h, an addition of 20 mL of SIF was made, followed by a pH adjustment to 6.8, and incubation continued under the same conditions for 4 h. Every 0.5 h, 2 mL of digestive solution was removed and substituted with an equal amount of SIF [34]. To the liquid with the microencapsulated samples, 6 mL of hexane was introduced. The mixture was thoroughly shaken, and then filtered. Two additional rounds of oil extraction from the remaining solids were performed. The oil-containing solvent phase was subsequently evaporated at 60 °C, and the remaining residue was dried at 105 °C until a constant weight was achieved. The release rate of deer oil is determined by the ratio of the deer oil content in the digestive solution to the total oil present in the microcapsules.

### 2.9. Statistical Analyses

Statistical analyses were performed using GraphPad Prism Software (version 9.0). The results are presented as means ± standard deviation (SD). One-way analysis of variance (ANOVA) was applied for comparing multiple groups, while a two-tailed unpaired Student’s *t*-test was used for comparing two groups. A *p* < 0.05 was considered statistically significant.

## 3. Results and Discussion

### 3.1. Deer Oil Fatty Acid Types and Content

**Figure 3:**
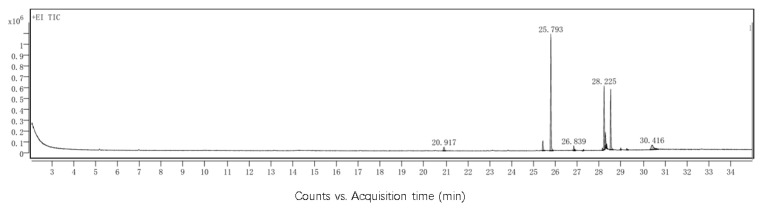
GC-MS analysis of deer oil components.

As shown in Figure 3, gas chromatography detected a total of seven fatty acids in deer oil. The four saturated fatty acids identified were myristic acid, palmitic acid, pearl fatty acid, and stearic acid, which together constituted 29.52% of the total fatty acid content. Additionally, two unsaturated fatty acids, linoleic acid and oleic acid, constituted 27.32% of the total, while one monounsaturated fatty acid, palmitoleic acid, had the highest content at 41.88%.

### 3.2. Optimization of the Complex Coacervation

#### 3.2.1. pH Optimization

**Figure 4:**
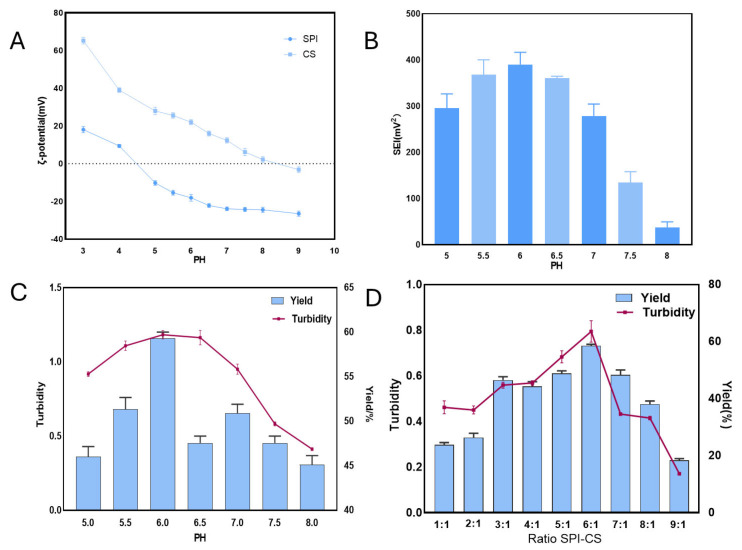
Optimization of pH and SPI/CS ratio (*n* = 3). (**A**) Zeta potential variation in SPI/CS at different pH. (**B**) SEI variation in SPI/CS at different pH. (**C**) Yield and turbidity of SPI/CS at different pH and ratios. (**D**) Yield and turbidity of SPI/CS at different ratios.

The development of soybean protein isolate–chitosan complexes is mainly governed by electrostatic forces [35]. The SEI between oppositely charged polyelectrolytes can be approximated by calculating the magnitude of the ζ potentials product for each polymer at various pH levels [36]. In Figure 4A, the ζ-potential pH profiles of SPI and CS are shown, revealing that they possess opposite charges within the pH range of 4.5 to 8.5, suggesting the formation of complex coacervates. Specifically, CS possesses a positive charge, and the observed decrease in ζ-potential with increasing pH is due to the deprotonation of its amino groups. Meanwhile, SPI carries a negative charge when the pH exceeds its pI (4.5). As shown in Figure 4B, the SEI–pH curves reveal that the likelihood of SPI–CS interaction is highest at pH 6.0 and lowest at pH 8.0. Figure 4C shows that the yield and turbidity results are consistent with these results, with a maximum at a pH of 6. Based on this finding, we identified pH 6.0 as the optimal condition for studying the binding between SPI and CS in complex coacervates.

#### 3.2.2. Optimization of SPI to CS Ratio

In the study of protein–polysaccharide composite materials, the ratio of biopolymers typically exerts a significant impact on the formation of complexes, primarily by modulating the charge balance between proteins and polysaccharides. The aggregation rate of the complexes is closely related to the charge balance. Studies have shown that the electrostatic interactions between protein and polysaccharide molecules play a decisive role in the stability and aggregation efficiency of the complexes [37]. When the charge balance is effectively controlled, polymer molecules can interact through electrostatic attraction, forming larger aggregates that increase both the turbidity and yield of the complexes. As shown in Figure 4D, the highest turbidity and yield (59.33 ± 0.67%) were observed at an SPI/CS ratio of 6:1. This phenomenon suggests that at this ratio, the interaction between SPI and CS reaches an optimal state, promoting the formation of stable complexes.

#### 3.2.3. Complex Condensations FT-IR Analysis

**Figure 5:**
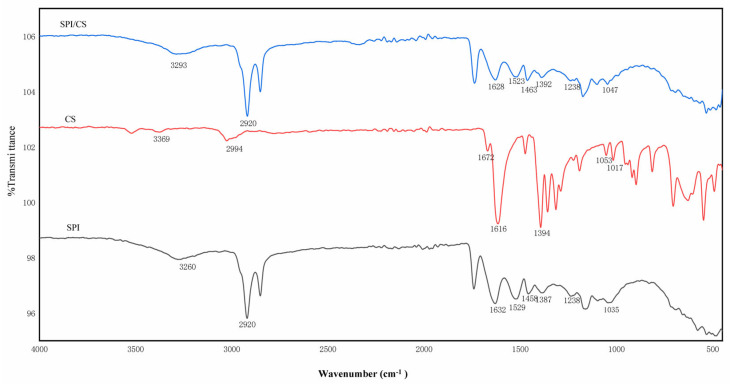
FTIR spectra of SPI, CS, SPI/CS.

The interaction between SPI and CS was examined using FTIR spectroscopy. The IR spectra of the individual walls without DO and the SPI and CS complexes are illustrated in Figure 5. The FTIR spectrum of CS shows characteristic peaks located at about 3369 cm^−1^ (O-H and N-H stretching vibrational absorption peaks) [38], around 2994 cm^−1^ (C-H stretching vibrational absorption peak) [39], 1672 cm^−1^ (characteristic of amide Ⅰ, C=O vibrational absorption peak) [40], 1615 cm^−1^ (characteristic of amide II, NH_2_ absorption peak), 1389 cm^−1^ (O-H and C-H vibrational absorption peaks), about 1017 cm^−1^ (C-O, vibrational absorption peak), and 1047 cm^−1^ (C-O-C vibrational absorption peak) [41]. The FTIR spectra of the SPI show distinctive peaks at about 3262 cm^−1^ (O-H stretching with N-H stretching vibration), 2920 cm^−1^ (CH_2_ asymmetric stretching) [42], 1628 cm^−1^ (amide I, C=O stretching) [43], 1524 cm^−1^ (amide II, N-H bending vibration) [44], 1461 cm^−1^ (C-H bending vibration), 1389 cm^−1^ (C-N and amide III with N-H vibrations), 1238 cm^−1^ (C-N stretching and N-H bending vibrations), and 1100 cm^−1^ (C-O stretching) [42].

In the FT-IR spectra of the SPI/CS complexes (Figure 5), the amide I, II, and III bands of SPI were shifted to 1628 cm^−1^, 1523 cm^−1^, and 1392 cm^−1^, respectively. The disappearance of the characteristic band of CS at 1616 cm^−1^ (NH3+ group) confirmed the presence of electrostatic interactions, leading to the complexation of SPI and CS [45]. Additionally, the bands between 3100 and 3500 cm^−1^ indicate the formation and alteration of hydrogen bonds. In the infrared spectra of SPI/CS, the absorption peak at 3265 cm^−1^ (O-H stretching with N-H stretching vibration) of SPI shifts to 3293 cm^−1^, suggesting that the interactions between CS and SPI are also associated with hydrogen bonding [46].

### 3.3. Optimization and Characterization of Deer Oil Microcapsules

#### 3.3.1. Optimization of the Core-to-Wall Ratio and the Overall Concentration of Wall Material in Deer Oil Microcapsules

**Figure 6:**
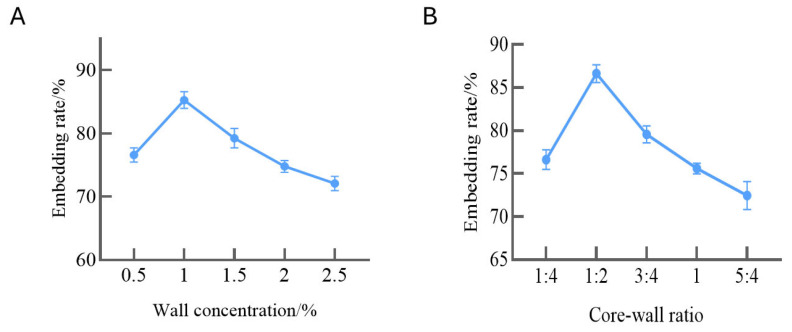
(**A**) Embedding rate of SPI/CS/DO at different wall concentrations. (**B**) Embedding rate of SPI/CS/DO at different core-to-wall ratios.

To identify the ideal wall material concentration, the impact of the overall wall material concentration on the efficiency of microcapsule encapsulation was studied. Figure 6A illustrates that the concentration of the wall material peaked at 1%, resulting in the highest embedding rate. The effect of the core-to-wall ratio on microcapsule encapsulation efficiency was examined, and as shown in Figure 6B, the core-to-wall ratio was highest at a 1:2 ratio with 85.28 ± 1.308%.

#### 3.3.2. Deer Oil Microcapsules FT-IR Analysis

**Figure 7:**
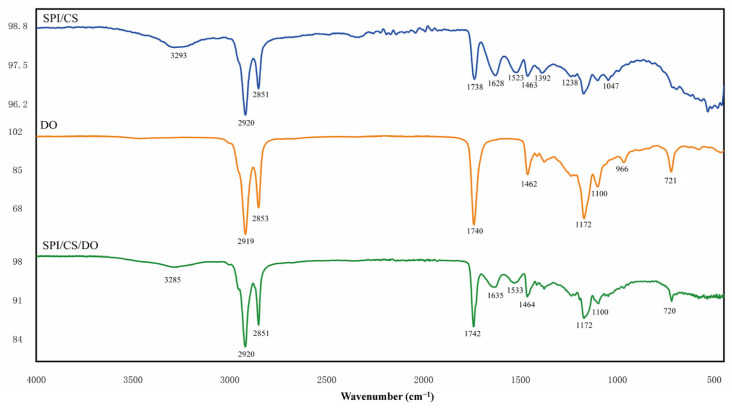
FTIR spectra of SPI/CS, SPI/CS/DO, DO.

Figure 7 displays the FTIR spectrum of DO, which includes the C-H stretching vibrations at 2919 cm^−1^ and 2853 cm^−1^, the C=O stretching vibration at 1740 cm^−1^, the C-H bending vibration at 1462 cm^−1^, and the C-O absorptions at 1172 cm^−1^ and 1100 cm^−1^. The absorption peak at 966 cm^−1^ corresponds to the out-of-plane bending vibration of -HC=CH- (trans), while the absorption peaks at 721 cm^−1^ are attributed to the bending vibrations of -(CH_2_)n and -HC=CH- (cis) [47]. The absorption bands observed at 1172 cm^−1^ and 1100 cm^−1^ correspond to the out-of-plane bending vibrations of the -HC=CH- (cis) group.

The absorption peaks in the infrared SPI/CS/DO spectrograms changed compared to DO. The results indicate that most of DO’s characteristic absorption peaks either vanished or weakened in the IR spectra of SPI/CS/DO. This might be caused by the constrained vibrational motion of DO molecules when bound with SPI/CS, suggesting that DO was encapsulated within the SPI/CS complex. For instance, the distinct absorption peak at 966 cm^−1^ in DO’s IR spectrum disappears in the IR spectrum of SPI/CS/DO, and the intensity of the absorption peak at 721 cm^−1^ is greatly reduced.

#### 3.3.3. Microstructure of Deer Oil Microcapsules

**Figure 8:**
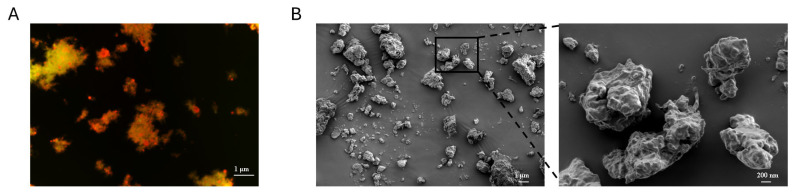
(**A**) CLSM images showing the oil phase (highlighted in yellow) and SPI/CS (highlighted in red), with the scale bar set to 1 μm. (**B**) SEM images of DO-loaded microcapsules, with scale bars of 200 μm and 50 μm shown in the figure.

Orthogonal fluorescence microscopy was employed to examine the formation of condensates involving SPI, CS, and DO, and to assess whether the oil droplets were encapsulated within these complexes. The pictures in Figure 8A illustrate how SPI and CS interactions led to the development of coalescent worm-like structures, consistent with the findings of Timilsena [48], Bakry et al. [49], and Feifan Li et al. [50]. Additionally, the coalescents were observed surrounding the oil droplets, indicating successful encapsulation of the oil droplets within the microcapsules.

Figure 8B presents the structural features of the freeze-dried microcapsules. All the microcapsules formed aggregates, displaying a rough and uneven structure, a typical occurrence during the freeze-drying process [51,52]. Furthermore, the microcapsules’ surface appeared intact without any visible fractures or gaps, suggesting sufficient interaction between SPI and CS to support microcapsule development.

#### 3.3.4. Thermal Stability of Deer Oil Microcapsules

**Figure 9:**
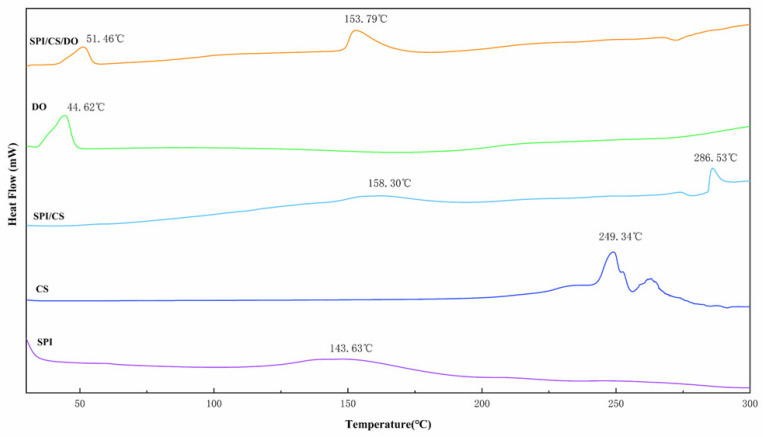
DSC spectra of SPI, CS, SPI/CS, DO, SPI/CS/DO.

The heat stability of microcapsules is evaluated with the DSC technique [53]. The thermal profiles of SPI, CS, SPI/CS, DO, and SPI/CS/DO are shown in Figure 9. The thermal spectra of SPI displayed a broad heat absorption peak within the range of 117.38–186.15 °C, with a peak temperature of 143.63 °C, corresponding to the evaporation of structural water. The peak temperature of heat absorption of CS was 249.34 °C, which represented the decomposition of the sugar chain of CS. There were two heat absorption peaks after the complex coacervate of SPI and CS, with peak temperatures at 158.30 °C and 286.53 °C, respectively, indicating that the complex coacervate increased the stability of SPI and CS [54]. The thermal profile of DO showed a heat absorption peak at 44.62 °C, indicating the melting point of DO. During the heating process, the SPI/CS/DO microcapsules experienced two phase transitions, occurring at peak temperatures of 51.46 °C and 153.79 °C, respectively. The initial phase transition corresponded to the melting of DO, indicating that DO’s melting temperature increased from 44.62 °C to 51.46 °C after encapsulation. The subsequent phase transition arises from the breakdown of SPI and CS, which manifests as small and narrow heat absorption peaks due to the formation of new solid network structures. The subsequent phase transition arises from the breakdown of SPI and CS, manifesting as small and narrow heat absorption peaks due to the formation of new solid network structures.

#### 3.3.5. Physical Properties of Deer Oil Microcapsules

**Figure 10:**
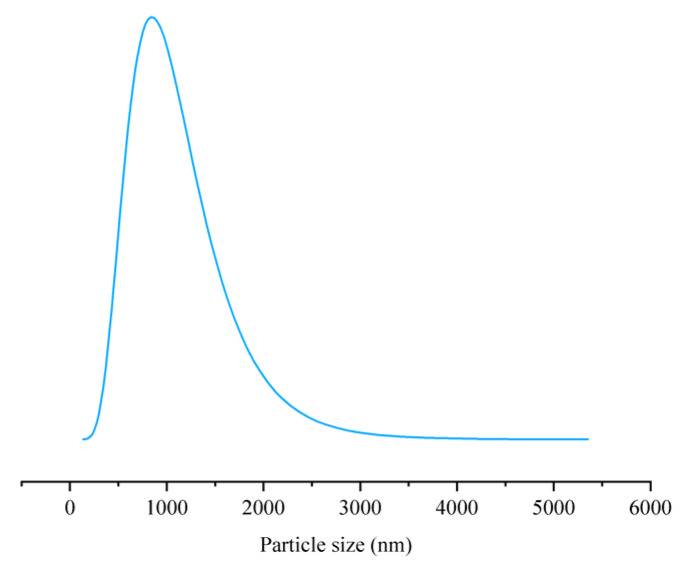
Deer oil microcapsules particle size distribution.

The distribution of particle sizes in deer oil microcapsules prepared under optimal conditions was examined, as illustrated in Figure 10 and Table 1. The microcapsules displayed a single peak distribution in particle size, with an average size of 2026 ± 183.5 nm, Indicating that the microcapsules produced in optimal conditions maintained a fairly consistent size. The PDI (Polydispersity Index) reflects the uniformity in particle size distribution; lower PDI values signify a more uniform and concentrated distribution [55]. As shown in Table 1, the PDI value of deer oil microcapsules was 0.287 ± 0.018, indicating a highly uniform size distribution.

Moisture content is an essential parameter for assessing microcapsules’ shelf life. The moisture level in the SPI/CS/DO microcapsules was under 5% (Table 1), indicating that the likelihood of microbial contamination in the microcapsules was reduced. The hygroscopicity influences the stability of the preservation of the core substance (deer oil). The microcapsules exhibited hygroscopicity (8.76%) after 7 days of storage. This can be attributed to the reduced availability of water binding sites due to the stronger electrostatic interactions between SPI and CS, resulting in a decrease in the water retention capacity of the microencapsulated powder. Similar findings were reported by Pereira et al. [56]. Comparable results were observed, demonstrating that microcapsules formulated with SPI/maltodextrin/pectin exhibited lower hygroscopicity compared to those prepared with SPI/maltodextrin. This variation is likely due to the stronger electrostatic interactions between SPI and pectin, which indicates that microcapsules made with SPI and CS show a reduced capacity to absorb moisture from the air, thereby enhancing their resistance to quality degradation in humid environments.

The packing density of the powder was about 0.334 ± 0.011 g.mL^−1^, which was similar to the encapsulated flaxseed oil [57], oregano essential oil [58], and gac oil [59]. A high packing density is advantageous for transporting and storing powders, as a higher packing density reduces the volume required for storage.

The ability of powders to flow is crucial for ensuring the efficiency of industrial activities such as their storage, compaction, and transportation [60]; these properties can be characterized by the angle of repose as well as by the Carr Index (CI) and the Hausner ratio (HR). The Carr Index allows the classification of powder mobility as “very good” (CI < 15%), “good” (15 < CI < 20%), “fair” (20 < CI < 35%), “poor” (35 < CI < 45%), “good” (35 < CI < 45%), “poor” (35 < CI < 45%), and “abysmal” (CI > 45%). Based on the Hausner ratio, the cohesion was categorized as “low” (HR < 1.2), “moderate” (HR < 1.4 < 1.2), and “high” (HR > 1.4) [61]. The angle of repose (θ) reflects “good flow” (<30°), “good” (31 < θ < 35°), “fair” (36 < θ < 40°), “passable but hangable” (41< θ < 45°), “poor that must be stirred or vibrated” (46 < θ <55°), and “inferior flow” (θ > 56°) [62]. As shown in Table 1, the microencapsulated powder had a CI of 14.396% and an angle of repose of 28.66 ± 1.305°, indicating a very good powder flow, and an HR of 1.168165468, indicating a low cohesion. 

### 3.4. Microencapsulated Oxidative Stability

**Figure 11:**
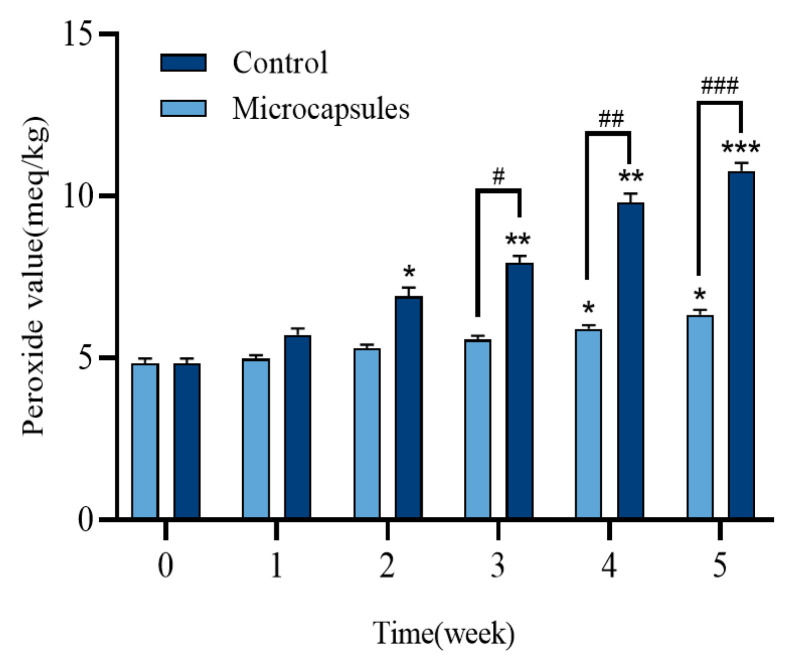
Oxidative stability of encapsulated and unencapsulated cores during storage. * *p* < 0.05, ** *p* < 0.01, *** *p* < 0.001, Control and Microcapsules groups compared with their respective initial; # *p* < 0.05, ## *p* < 0.01, ### *p* < 0.001, Microcapsules group compared with Control group.

In this study, the primary peroxide values were assessed to determine the oxidative stability of the encapsulated cores. Figure 11 illustrates the peroxide values of both the encapsulated core and the control (DO) stored at 37 °C for 5 weeks. After 5 weeks of storage at 37 °C, the peroxide level of the encapsulated core material remained under 6 meq O2/kg fat. In comparison, after 5 weeks of storage, the peroxide value of the control group significantly elevated. Additionally, After 2 weeks of storage, the peroxide value in the control group was notably higher than in the microencapsulated group (*p* < 0.05). The reduced peroxide concentration in the microcapsules suggests that the composite cohesive structure effectively resists oxidation by restricting gas diffusion [63].

### 3.5. The Release Behavior of SPI/CS/DO Microcapsules in Simulated Gastrointestinal Digestion SPI/CS/DO

**Figure 12:**
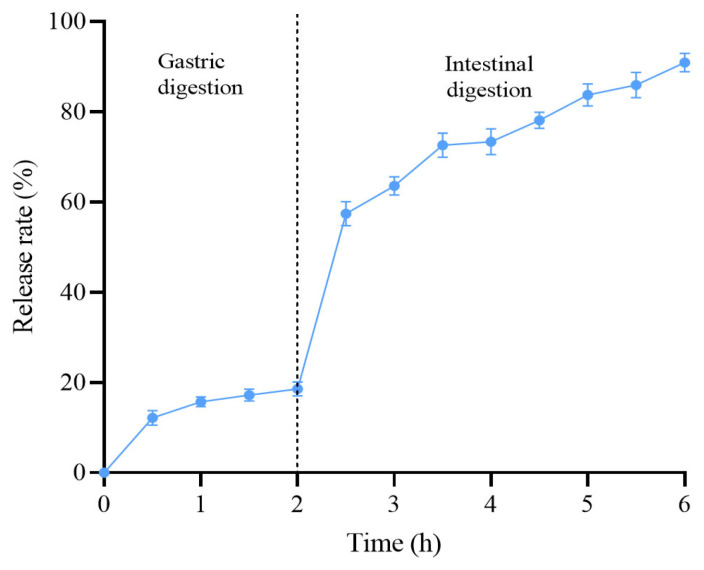
Simulates the cumulative release of DO from microcapsules during digestion.

As shown in Figure 12, during the SGF stage, all samples showed a gradual increase in their values. The observed phenomenon is likely due to the marked decrease in electrostatic interactions within the SPI/CS complex under highly acidic conditions, where SPI is nearly neutral and CS is positively charged (Figure 4A,B). Consequently, a minor disruption in the SPI/CS complex structure led to a gradual and prolonged release of DO. These findings indicate that the SPI/CS/DO microcapsules are resistant to the harsh acidic environment of SGF. The decreased oil release observed could be attributed to the clustering or coalescence of emulsion droplets upon contact with gastric enzymes in acidic conditions [34]. As anticipated, the combination of wall materials safeguarded the bioactive agent during its transit through the human stomach. During the SIF phase, all samples showed a sharp increase in release rate before the release rate reached equilibrium. This was attributed to the rapid destruction of the pod walls formed by the SPI/CS complex by trypsin, which led to the loss of pod structure and accompanying oil release [64]. Furthermore, the relatively small particle size of the microcapsules resulted in an increased contact surface area during enzymatic hydrolysis. Thus, the SPI/CS complex was successfully disassembled, resulting in a sustained release of DO. Our findings are consistent with those of Karaca et al. [64], who investigated the release characteristics of flaxseed oil microcapsules in vitro, employing a wall matrix composed of chickpea or lentil protein isolate combined with maltodextrin. Around 37.6% of the flaxseed oil encapsulated in the microcapsules was released after 2 h under SGF conditions, and approximately 46.6% was released after 3 h under SIF conditions.

## 4. Conclusions

In this study, we first utilized SPI and CS for complex coacervates via electrostatic attraction and successfully prepared DO-loaded microcapsules. At pH 6, the microcapsules with a 6:1 SPI/CS ratio and a 1:2 core-to-wall ratio achieved the highest encapsulation efficiency for deer oil (85.28 ± 1.308%). The SEM and CLSM analyses demonstrated that DO was effectively encapsulated within the microcapsules, and the encapsulated core material had good oxidative stability. Results from the simulated in vitro digestion indicated that the DO-encapsulated microcapsules showed high stability in gastric acid environments and would continuously release their contents in the SIF. This research offers a pragmatic method for utilizing DO in food, pharmaceuticals, and various other industries. It also delivers essential insights for the innovation and development of new functional food ingredients incorporating deer oil.

## Figures and Tables

**Table 1:** Physicochemical properties of deer oil microcapsules.

Physical Property	SPI/CS/DO
Embeddedness/%	85.28 ± 1.308
Particle size/nm	2026 ± 183.5
PDI	0.287 ± 0.018
Zeta	22.78 ± 4.88
Water content/%	1.3
Absorbent property	8.76
Bulk density	0.334 ± 0.011
Knockdown density	0.390 ± 0.009
Angle of repose/°	28.66 ± 1.305
Carr Index	14.396
Hausner ratio	1.168

## Data Availability

The original contributions presented in the study are included in the article, further inquiries can be directed to the corresponding authors.
